# Stress Fracture and Nonunion of Coronoid Process in a Gymnast

**DOI:** 10.1155/2016/9172483

**Published:** 2016-06-23

**Authors:** T. Hetling, P. Bourban, B. Gojanovic

**Affiliations:** ^1^Swiss Federal Institute for Sport (BASPO), Swiss Olympic Medical Center, 2532 Magglingen, Switzerland; ^2^Swiss Sportclinic, 3000 Bern, Switzerland; ^3^La Tour Sports Medicine, Swiss Olympic Medical Center, Hôpital de La Tour, Meyrin, 1217 Geneva, Switzerland; ^4^Sports Medicine, Department for Human Locomotion (DAL), Lausanne University and Hospital, 1011 Lausanne, Switzerland

## Abstract

*Background*. Gymnasts have high mechanical loading forces of up to 14 times body weight. Overuse lesions are typical in wrists and stress fractures in the olecranon, while isolated fractures of the coronoid process are uncommon. We present a case of retraumatized nonunion stress fracture of the ulnar coronoid process.* Case Description*. A 19-year-old gymnast presented with elbow pain after training. Imaging confirmed an old fracture of the coronoid process. We describe a 6-month multiphase return to competition rehabilitation program, which allowed him to compete pain-freely.* Literature Review*. Acute and overuse injuries in gymnasts are known but no nonunion of the coronoid process has been described before. Only one case of stress fracture of coronoid process in a gymnast was reported.* Purpose and Clinical Relevance*. We could successfully and conservatively return to sport a reactivated nonunion of a stress fracture of the coronoid process.

## 1. Introduction

Artistic gymnastics is a sport with high mechanical loading forces (up to 14 times body weight) impacting the whole body. Unsurprisingly, this leads to overuse/stress lesions in the lower and upper extremities (shoulder, elbow, and wrist). Stress fractures have been reported in the radius and olecranon [1–3], whereas isolated coronoid process fractures are uncommon. We present a case of a retraumatized nonunion of ulnar coronoid process stress fracture.

The coronoid process usually fractures in high energy trauma of the elbow (within the “terrible triad” of posterolateral dislocation, radial head, and coronoid fracture) [1–3]. The mechanism of coronoid fracture involves twisting with hyperflexion or hyperextension [4]. In gymnasts, axial loading causes a varus posteromedial rotational force, with the medial trochlea riding up onto the anteromedial aspect of the coronoid process. This leads to shearing and overuse and can be acutely associated with lateral collateral ligament (LCL) injury. Both structures have an important role in varus stability [5].

Coronoid fractures are of three types as described by Regan and Morrey and depend on the location of the fracture along the coronoid on lateral radiographs [3] and a new classification system by O'Driscoll et al. based on the anatomic location of coronoid fragments [2]. Important structures attach onto the coronoid process, such as the tendon of brachialis muscle, the anteriro joint capsule, and the anterior band of the media collateral ligament (AMCL). Most important is the sublime tubercle (insertion of AMCL), a key elbow stabilizer. The coronoid process is hence the most critical bony stabilizer of the elbow joint [5].

## 2. Case Report

**Figure 1:**
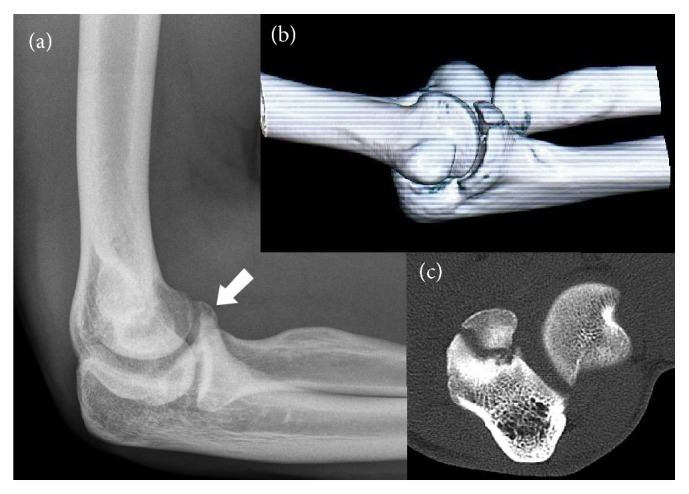
Imaging of the elbow at diagnosis. (a) X-ray: irregularity of the anterior elbow joint. (b) Three-dimensional CT-scan reconstruction of ancient coronoid process fracture. (c) Axial view of elbow on CT-scan.

A 19-year-old elite gymnast presented with right elbow pain for 4 weeks, without recollection of a provoking trauma. Pain appeared in full extension upon loading in gymnastics training. Clinical examination revealed no instability upon valgus or varus stress and pain was provoked by end of range movements, which were limited in flexion (20° less than left elbow). Radiographs showed an irregularity in the anterior elbow joint, potentially corresponding to a free fragment or fracture of the coronoid process. MRI and CT-scan (Figure 1) confirmed a nonunion of a coronoid process fracture Type 2 by O'Driscoll et al. (most probably an ignored stress fracture). The olecranon showed increased signal corresponding to bone bruise, and fluid was present in the joint. The fragment was not dislocated and the gap of the joint line was 3.7 mm. We interpreted the injury as a retraumatization of a coronoid nonunion.

We describe the rehabilitation of this unusual lesion in gymnastics, which we divided into five phases discussed between the physician, physiotherapist, gymnastics coach, and the athlete himself.

### 2.1. Phase 1: Weeks 1 to 4—Unloading and Passive Mobilization

This phase consisted of initial immobilization and unloading in the first 2 weeks, with passive mobilization in weeks 3 and 4. After this phase, a repeat X-ray confirmed the absence of dislocation.

### 2.2. Phase 2: Weeks 5 to 8—Prepare to Train

It consists of progressive active mobilization without resistance to end of range, initially only for elbow extensors. Clinically, the athlete complained of decreasing light pain in hyperextension and full flexion. MRI at 8 weeks after this phase of active mobilization showed no secondary dislocation and a significant reduction of the edema in the bone bruise area.

### 2.3. Phase 3: Weeks 9 to 12—Prepare to Perform

Loading and weight bearing were slowly introduced in training. In physiotherapy, the load was controlled and raised regularly according to pain, which was continuously monitored to ascertain reduction while load was increasing (dumbbells, press-ups). Throughout all exercises, varus and valgus stress were controlled. Full symmetrical ROM with a stable joint was achieved at the end of this phase.

### 2.4. Phase 4: Weeks 13 to 24—Train to Compete

**Figure 2:**
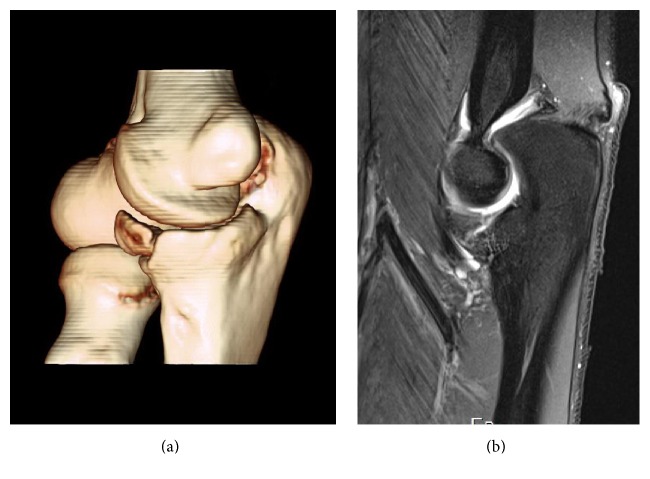
Follow-up imaging studies at 4 and 6 months after diagnosis. (a) Three-dimensional CT-scan reconstruction of coronoid process nonunion: no callus formation and no displacement. (b) Elbow MRI: nonunion and lack of edema or joint effusion.

It consists of specific gymnastics training (beginning with floor and pommel horse, before introducing hanging elements and vaults) with a maximum authorized pain from 2 to 3 (on a 1–10 visual analogic scale). A third MRI at 4 months (Figure 2(b)), 2 weeks into this phase, showed only limited intra-articular fluid without bone edema. The nonunion was unchanged (gap remained identical).

### 2.5. Phase 5: After 6 Months—Return to Competition

At 6 months, the gymnast was pain-free and able to complete all requirements for competition on floor, vault, pommel horse, and high and parallel bars. At this point a CT-scan was repeated and could show similarly to the initial pictures no change in nonunion and fragment position (Figure 2(a)). No callus bone appeared, although full athletic function was recovered.

## 3. Discussion

We report for the first time a reactivated nonunion isolated stress fracture of the coronoid process in an elite artistic gymnast, for which we successfully conducted a 5-step conservative rehabilitation to allow for full return to competition at international level.

Many traumatic and overuse injuries have been described in gymnastics, but nonunion of the coronoid process seems to be a rare occurrence. Rettig and Mathis described one case of coronoid stress fracture, which healed to union over more than 6 months and required electrical bone stimulation [6]. No case of isolated nonunion of a stress fracture of the coronoid process has been reported, although two cases of olecranon stress fractures and nonunions in gymnasts have been described [7]. The high load and weight bearing stress required by elite gymnasts place them at high risk for overuse injuries of the upper extremity. Greatest load at the coronoid process is generated with axial loading between neutral and 15° of hyperextension [8].

Although we could not see any changes in ossification and dislocation, a progressive rehabilitation and strengthening program allowed for return to competition, and this should be considered in cases where the elbow is stable and the sublime tubercle is not involved [9]. The type of fracture we encountered is rarely found in gymnasts, in whom avascular necrosis of the capitellar epiphysis, osteochondrosis dissecans, or changes of the radial head are rather observed. It is usually reported in baseball pitchers and throwers, but we recommend it should be considered in gymnasts as well [10].
